# A lymphadenectomy and irradiation-induced rat lymphedema model exhibits persistent dermal backflow and region-dependent fibrosis-associated remodeling

**DOI:** 10.3389/fcell.2026.1901231

**Published:** 2026-08-31

**Authors:** Hayeong Cho, Jae Hoon Jeong, Joseph Kyu-Hyung Park, Sung-Hwan Yoon, Chan Yeong Heo, Yun‐Sang Lee, Yujin Myung

**Affiliations:** 1 Department of Plastic and Reconstructive Surgery, Seoul National University Bundang Hospital, Seongnam, Democratic People’s Republic of Korea; 2 Department of Health Science and Technology, Graduate School of Convergence Science and Technology, Seoul National University, Seoul, Democratic People’s Republic of Korea; 3 Department of Nuclear Medicine, Seoul National University College of Medicine, Seoul, Democratic People’s Republic of Korea; 4 Department of Molecular Medicine and Biopharmaceutical Sciences, Graduate School of Convergence Science and Technology, Seoul National University, Seoul, Democratic People’s Republic of Korea; 5 Department of Plastic and Reconstructive Surgery, Seoul National University College of Medicine, Seoul, Republic of Korea

**Keywords:** alpha-smooth muscle actin, collagen III, dermal backflow, indocyanine green lymphography, rat hindlimb model, secondary lymphedema

## Abstract

**Background and Aims:**

Secondary lymphedema is characterized by persistent lymphatic dysfunction and tissue remodeling after oncological treatment. Existing rodent models often show spontaneous recovery and are evaluated using limited endpoints. We aimed to establish a persistent rat hindlimb model and characterize its temporal and spatial progression through integrated functional, histological, tissue-level, and circulating assessments.

**Methods:**

Secondary lymphedema is characterized by persistent lymphatic dysfunction and tissue remodeling after oncological treatment. Existing rodent models often show spontaneous recovery and are evaluated using limited endpoints. We aimed to establish a persistent rat hindlimb model and characterize its temporal and spatial progression through integrated functional, histological, tissue-level, and circulating assessments.

**Results:**

Both groups exhibited severe early dermal backflow. By weeks 4–6, control hindlimbs showed partial restoration of organized linear drainage, whereas lymphedema hindlimbs retained significantly higher dermal backflow scores and persistent unorganized drainage. At week 6, all lymphedema hindlimbs remained within Type IIIa or IIIb categories. Dermal thickness was greater in lymphedema hindlimbs at both anatomical sites. Immunofluorescence showed directionally higher collagen III and α-SMA signals in subsets of paired lymphedema tissues; however, none of the comparisons reached statistical significance, and substantial inter-animal variability was observed. Circulating α-SMA was significantly higher in the lymphedema group at days 1 and 7, whereas PIIINP was numerically higher at days 7 and 15 without significant between-group differences.

**Conclusion:**

Radical lymphadenectomy combined with circumferential skin resection and localized irradiation produced persistent lymphatic impairment and sustained regional dermal remodeling. Collagen III and α-SMA immunofluorescence provided additional evidence of heterogeneous fibrosis-associated tissue changes. Circulating α-SMA may provide a complementary systemic readout, whereas PIIINP remains exploratory. This multimodal model offers a preclinical framework for studying secondary lymphedema progression and evaluating therapeutic strategies.

## 1 Introduction

Lymphedema is a chronic condition characterized by insufficient lymphatic transport caused by lymphatic hypoplasia, dysfunction, or structural obstruction. The resulting accumulation of protein-rich interstitial fluid leads to progressive swelling and physical symptoms, including heaviness, tightness, and pain (Cueni and Detmar, 2006; Finnane et al., 2015). Secondary or acquired lymphedema, the most common form of the disease, typically results from structural damage to the lymphatic vascular system caused by disease, trauma, or surgical intervention (Detmar and Hirakawa, 2002; Grada and Phillips, 2017). This localized impairment not only causes fluid accumulation but also alters regional immune homeostasis and tissue structure. Secondary lymphedema is a frequent and debilitating complication among cancer survivors. It is particularly prevalent following breast cancer treatment, with reported incidence rates ranging from 6% to more than 70%, depending on the extent of surgery, use of radiation therapy, diagnostic criteria, and duration of follow-up (Cormier et al., 2010).

**FIGURE 1:**
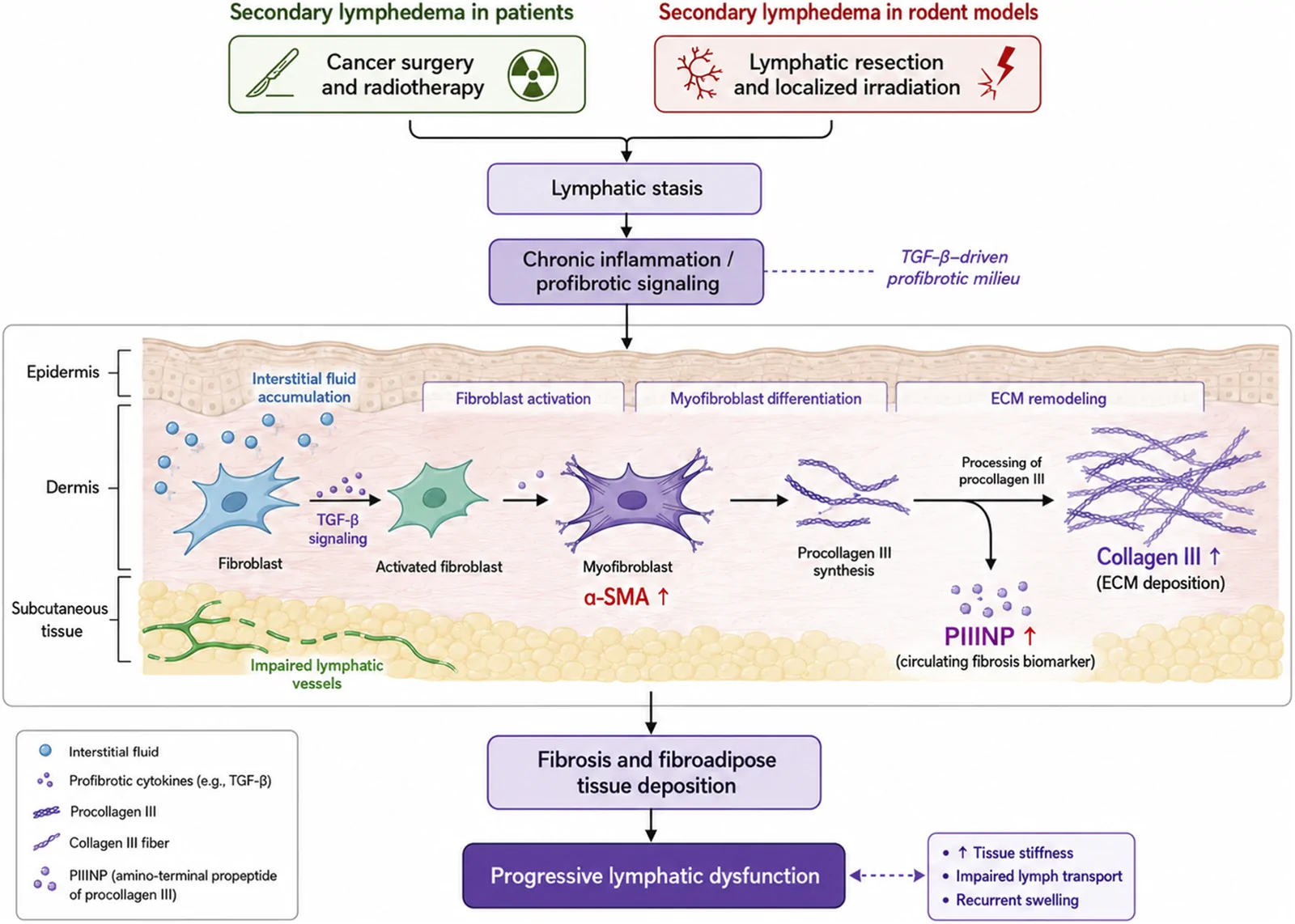
Schematic overview of secondary lymphedema pathophysiology in patients and the rat model. Cancer surgery and radiotherapy in patients, and radical lymphadenectomy, circumferential skin resection, and localized irradiation in the rat model, disrupt lymphatic drainage and induce lymphatic stasis. Persistent stasis promotes chronic inflammation and profibrotic signaling, leading to fibroblast activation, α-SMA-associated myofibroblast differentiation, collagen III deposition, and extracellular matrix remodeling. PIIINP, released during type III collagen formation, is shown as an exploratory circulating marker of collagen-associated remodeling. Progressive fibrosis and tissue stiffness may further impair lymphatic transport and sustain lymphatic dysfunction. The schematic represents a proposed biological framework; not all depicted pathways were directly assessed in this study. Abbreviations: α-SMA, alpha-smooth muscle actin; ECM, extracellular matrix; PIIINP, N-terminal propeptide of type III procollagen; TGF-β, transforming growth factor beta.

The development of secondary lymphedema following oncological treatment is primarily driven by sustained impairment of lymphatic drainage. Surgical procedures such as axillary or inguinal lymph node dissection, which are commonly performed in patients with breast cancer, melanoma, and other malignancies, disrupt the regional lymphatic network. This injury may be further aggravated by regional nodal or chest wall irradiation, which increases the risk of lymphedema by damaging lymphatic vessels and promoting soft-tissue fibrosis (Figure 1) (Grada and Phillips, 2017; Cormier et al., 2010; Hayes et al., 2008; Shaitelman et al., 2017). Despite the substantial burden of the disease, conservative physiotherapy remains the principal treatment for many patients. Emerging microsurgical techniques, including lymphovenous anastomosis and vascularized lymph node transfer, have shown clinical potential; however, robust evidence regarding patient selection, long-term efficacy, and comparative therapeutic outcomes remains limited (Koshima et al., 2000; Becker et al., 2012). Therefore, reproducible and translationally relevant animal models are needed to clarify disease progression and rigorously evaluate surgical, pharmacological, and regenerative therapies.

Various animal models of secondary lymphedema have been developed to reproduce lymphatic dysfunction following surgery or irradiation (Frueh et al., 2016). However, spontaneous lymphatic regeneration and collateral drainage can result in partial or complete recovery, particularly in rodent models. In addition, many studies rely primarily on limb-volume measurements, endpoint lymphatic imaging, or histological assessment at a limited number of time points. Such approaches may not fully distinguish transient postoperative edema from persistent lymphatic dysfunction or capture the spatial and temporal heterogeneity of tissue remodeling. Histological analyses are also frequently limited to dermal thickness or a restricted panel of tissue markers, while local fibrosis-associated changes and circulating responses are rarely evaluated within a common experimental framework. A longitudinal and multimodal approach combining functional imaging, anatomically defined histology, local molecular analysis, and systemic measurements may therefore provide a more comprehensive assessment of model development.

Secondary lymphedema is not solely a disorder of fluid accumulation but is also characterized by progressive inflammatory and fibrotic tissue remodeling (Rockson, 2018). Persistent lymphatic stasis promotes chronic immune-cell infiltration, adipose deposition, fibroblast activation, and extracellular matrix accumulation (Sung et al., 2022; Younesi et al., 2024; Zoon et al., 2017). TGF-β1 signaling has been implicated in this process by promoting fibroblast activation and extracellular matrix deposition while inhibiting lymphatic regeneration. Increased TGF-β1 signaling and extracellular matrix expression have also been demonstrated in human lymphedematous tissue and experimental models of secondary lymphedema (Baik et al., 2022).

Activated fibroblasts can acquire a contractile myofibroblast phenotype characterized by increased expression of α-smooth muscle actin (α-SMA) and enhanced production of extracellular matrix proteins, including collagen (Will et al., 2024; Klingberg et al., 2013; Gibb et al., 2020) (Figure 1). α-SMA is therefore widely used as a tissue-level marker of myofibroblast-associated remodeling, although it is not specific to fibroblasts or lymphedema. Type III collagen is an important component of remodeling extracellular matrix, and increased type III collagen transcription has been reported in fibrotic skin lesions from patients with lower-extremity lymphedema (Karayi et al., 2020). These findings provide a biological rationale for evaluating local α-SMA and collagen III signals as complementary indicators of fibrosis-associated tissue remodeling.

The amino-terminal propeptide of type III procollagen (PIIINP) is released into the circulation during type III collagen synthesis and can be measured as a systemic marker of collagen III formation (Karsdal, 2019; Bhasin et al., 2009). Circulating α-SMA has also been investigated as a potential non-invasive fibrosis-associated marker in non-lymphatic diseases, including liver fibrosis (Cardoso-Lezama et al., 2024). However, neither circulating α-SMA nor PIIINP is established as a specific biomarker of lymphedema. Their concentrations may also be influenced by wound healing, surgical trauma, irradiation, and systemic extracellular matrix remodeling. For example, serum PIIINP has been associated with fibrotic remodeling following severe burn injury (Ulrich et al., 2003). Therefore, circulating α-SMA and PIIINP should be evaluated as exploratory systemic markers and interpreted together with direct measurements of regional lymphatic function and local tissue remodeling rather than as direct proxies for lymphedema-specific fibrosis.

To address these limitations, we established a rat hindlimb model of secondary lymphedema using a combined injury strategy consisting of radical lymphadenectomy, circumferential skin resection, and localized irradiation. Both the control group and lymphedema group underwent circumferential skin resection and irradiation, whereas radical lymphadenectomy was performed only in the lymphedema group, allowing the additional effects of extensive lymphatic ablation to be evaluated. We hypothesized that radical lymphadenectomy would prevent spontaneous recovery of lymphatic drainage and produce persistent regional tissue remodeling beyond the responses caused by skin resection and irradiation alone.

The model was evaluated using a multimodal and longitudinal framework. Functional lymphatic impairment was assessed by serial indocyanine green lymphography over 6 weeks using a clinically adapted dermal backflow classification system. Dermal structural changes were quantified in the ankle and footpad to evaluate anatomically distinct proximal and distal regions. Local fibrosis-associated remodeling was further assessed by immunofluorescence analysis of α-SMA and collagen III. In separate animal cohorts, circulating α-SMA and PIIINP were serially measured as exploratory systemic markers of tissue repair and extracellular matrix remodeling. By integrating functional imaging, regional histology, local fibrosis-associated markers, and circulating measurements, this study aimed to comprehensively characterize the temporal and spatial progression of secondary lymphedema and establish a preclinical framework for evaluating therapeutic responses.

## 2 Materials and methods

All experimental procedures involving animals were conducted in accordance with the Guide for the Care and Use of Laboratory Animals and were approved by the Institutional Animal Care and Use Committee (IACUC) of Seoul National University Bundang Hospital (IACUC No. BA-2403–387-009–06). The study was prepared in compliance with the ARRIVE guidelines. Male Sprague Dawley rats (6 weeks old) were obtained from ORIENT BIO (Seongnam, Republic of Korea) and housed under conditions consistent with the standards of the Association for Assessment and Accreditation of Laboratory Animal Care International system. Animals were maintained in a controlled environment with regulated temperature and lighting cycles.

### 2.1 Radical lymphadenectomy and circumferential skin resection

**FIGURE 2:**
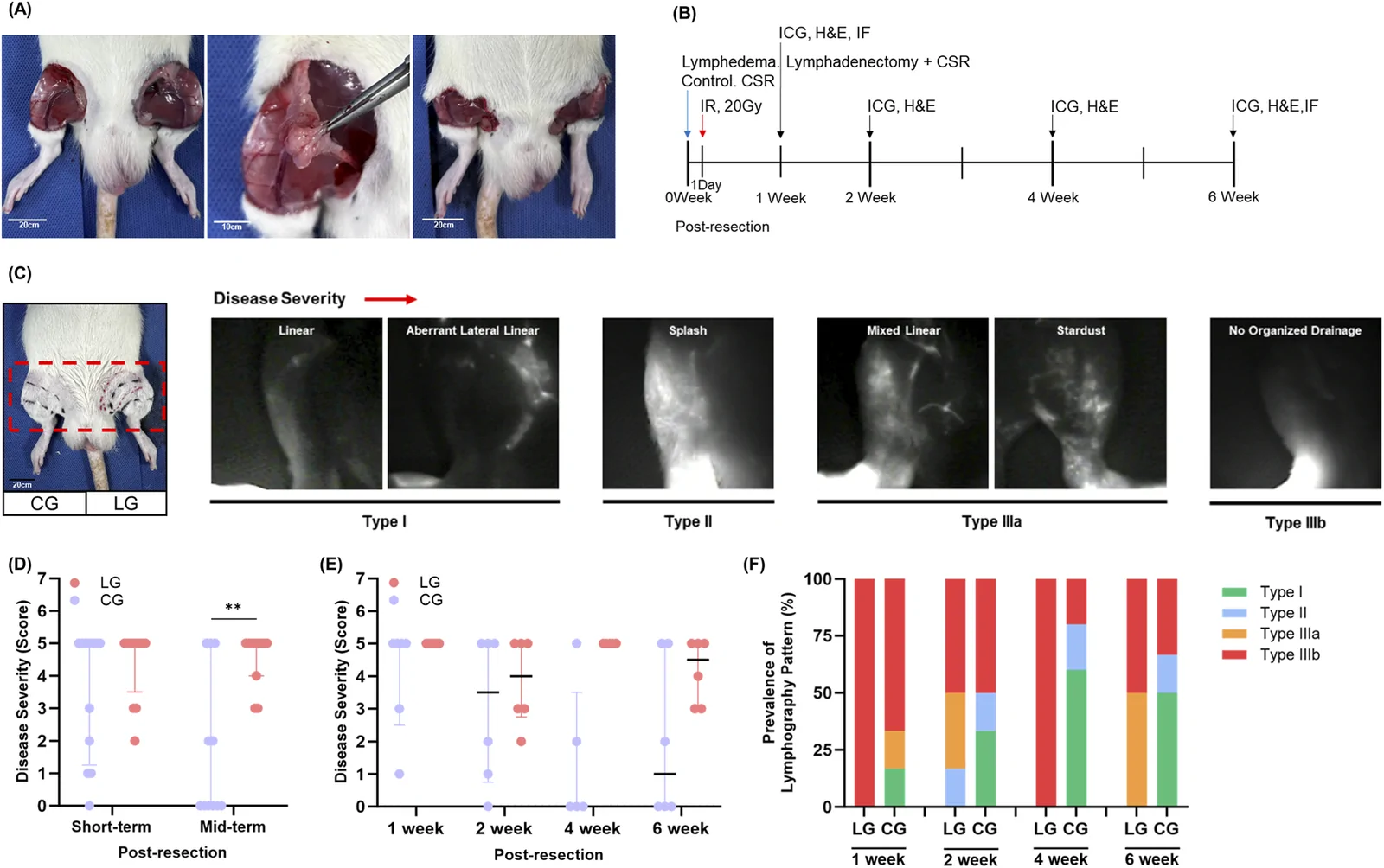
Longitudinal assessment and clinically adapted classification of lymphatic dysfunction using ICG lymphography **(A)** Illustration of the surgical procedures. A 1-cm-wide CSR was performed to disrupt superficial collateral lymphatic pathways. In the LG, the inguinal lymph node and surrounding deep fat pad were additionally resected, whereas these structures were preserved in the CG **(B)** Experimental timeline showing multimodal assessments performed at 1, 2, 4, and 6 weeks post-resection **(C)** Macroscopic view of the bilateral hindlimb model and representative ICG lymphography images showing the modified classification of lymphatic drainage patterns into four clinically adapted types, from Type I to Type IIIb, corresponding to an ordinal severity score of 0–5 **(D)** Phase-based comparison of lymphatic dysfunction scores during the early phase (weeks 1–2) and later phase (weeks 4–6). The early-phase analysis included 12 paired limb observations obtained from six animals assessed at weeks 1 and 2. The later-phase analysis included 11 paired limb observations obtained from five animals at week 4 and six animals at week 6. Comparisons between paired CG and LG hindlimbs were performed using the Wilcoxon matched-pairs signed-rank test (p < 0.01) **(E)** Longitudinal ordinal severity scores in the CG and LG at 1, 2, 4, and 6 weeks post-resection. Sample sizes were six paired animals at weeks 1, 2, and 6 and five paired animals at week 4. Data are presented as the median and interquartile range **(F)** Distribution of ICG lymphography pattern types over time. Severe Type IIIa and IIIb patterns persisted in the LG, whereas the CG progressively shifted toward Type I and II patterns. Sample sizes were six paired animals at weeks 1, 2, and 6 and five paired animals at week 4. Abbreviations: CG, control group; CSR, circumferential skin resection; ICG, indocyanine green; LG, lymphedema group.

**FIGURE 3:**
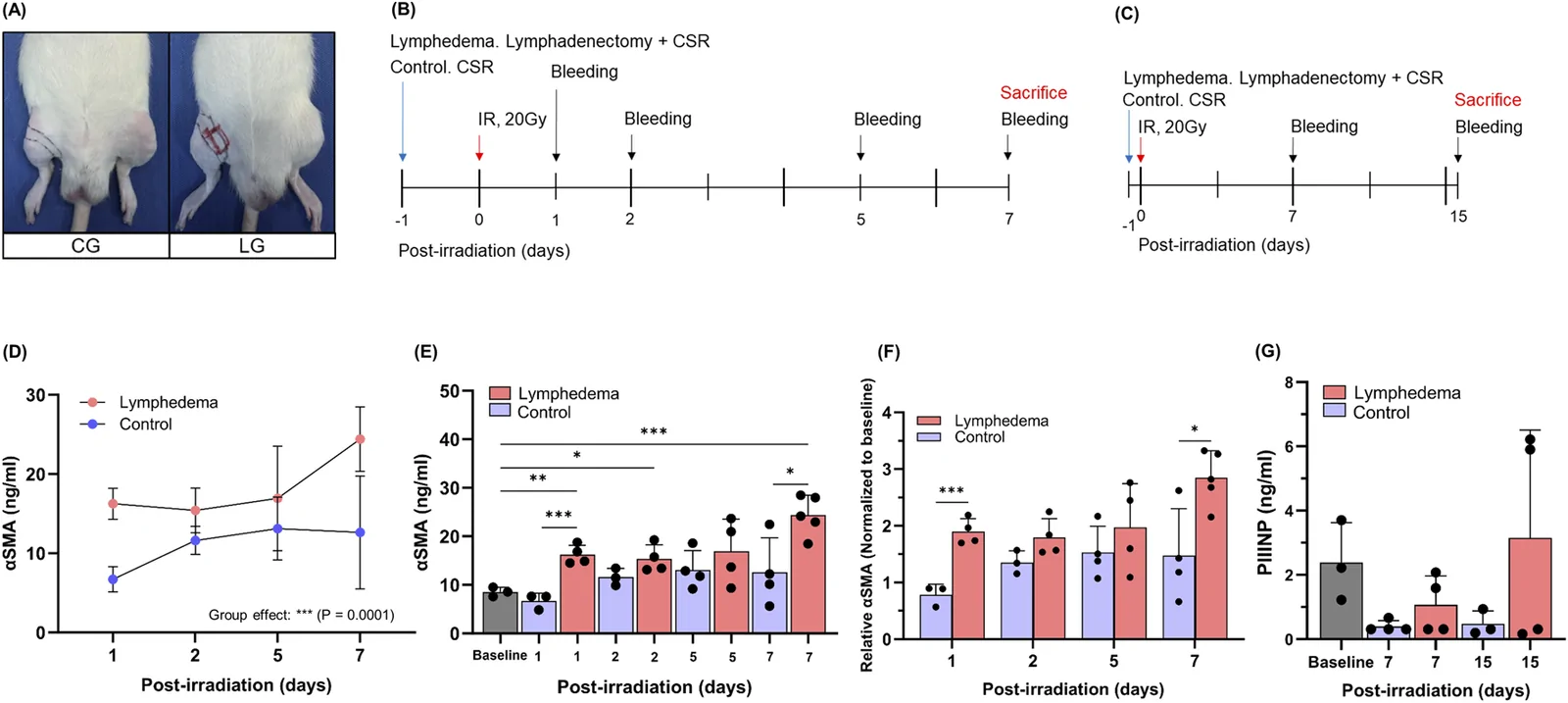
Longitudinal serum α-SMA and PIIINP profiles in unilateral control and lymphedema cohorts **(A)** Representative gross images of the unilateral CG and LG models. Separate animals were assigned to the CG and LG for group-specific serum analysis **(B,C)** Experimental timelines for serum α-SMA and PIIINP measurements **(D-F)** Longitudinal serum α-SMA profiles, absolute concentrations, and fold changes normalized to the preoperative baseline (baseline = 1.0). For α-SMA analysis, the baseline included n = 3 animals. Sample sizes for the CG were n = 3, 3, 4, and 4 at days 1, 2, 5, and 7, respectively, and those for the LG were n = 4, 4, 4, and 5, respectively **(G)** Serum PIIINP concentrations at days 7 and 15. Sample sizes were baseline, n = 3; day 7, CG n = 4 and LG n = 4; and day 15, CG n = 3 and LG n = 4. PIIINP levels were numerically higher in the LG, but the between-group differences were not statistically significant at day 7 (p = 0.2323) or day 15 (p = 0.2113). Values below the lower limit of detection were assigned 0.3125 ng/ mL, corresponding to LOD/2. LOD/2 substitution was applied to 2 of 4 LG and 3 of 4 CG samples at day 7, and 1 of 4 LG and 1 of 3 CG samples at day 15. Data are presented as the mean ± SD. Longitudinal α-SMA profiles were analyzed using a mixed-effects model, and individual between-group comparisons were performed using an unpaired Student's t-test or Welch's t-test, as appropriate. Statistical significance is indicated as *p < 0.05, **p < 0.01, and ***p < 0.001.. Abbreviations: α-SMA, alpha-smooth muscle actin; CG, control group; CSR, circumferential skin resection; LG, lymphedema group; LOD, lower limit of detection; PIIINP, N-terminal propeptide of type III procollagen.

Two experimental groups were established to evaluate the pathophysiological impact of lymphatic disruption: the Lymphedema Group (LG) and the Control Group (CG). For ICG lymphography, histological analyses, and immunofluorescence, one hindlimb in each rat was assigned to the lymphedema group, whereas the contralateral hindlimb served as the paired control limb (Figures 2A,C). For serum marker analyses, rats were randomly assigned to either the LG or CG, and each animal contributed one experimental hindlimb (Figure 3A).

All procedures were performed on 7-week-old rats under general anesthesia induced via intramuscular (i.m.) injection of a Zoletil (approx. 50 mg/kg) and Rompun (approx. 4.7 mg/kg) mixture (5:1 ratio). To induce chronic secondary lymphedema in the LG, a radical excision of the inguinal fat pad was performed. Anatomically, an incision was made to expose the superficial epigastric vein, and the entire fat pad encompassing the inguinal lymph node and the surrounding superficial and deep lymphatic networks was carefully dissected. Dissection was performed meticulously to minimize microvascular trauma, and any minor capillary bleeding was effectively controlled with gentle pressure using sterile gauze. Based on visual identification, the fat pad was completely removed down to the underlying skeletal muscle. This was performed in conjunction with a circumferential skin resection. To minimize collateral lymphatic regeneration, the upper and lower skin margins were anchored directly to the underlying muscular fascia using interrupted skin-to-fascia fixation sutures with 5-0 monofilament nylon (Ailee Co., Ltd., Busan, Republic of Korea), leaving a standardized 1-cm-wide circumferential skin defect (Figure 2A). In contrast, the CG underwent only the circumferential skin resection without deep lymphatic tissue excision. The detailed surgical technique for extended lymphadenectomy is demonstrated in Supplementary Video S1, and the step-by-step procedures and group allocations are illustrated in Figures 2A, 3A.

The open wounds were allowed to heal by secondary intention under daily clinical monitoring within a Specific Pathogen-Free facility, in accordance with the approved IACUC protocol. Postoperative animal condition was assessed daily based on general appearance, activity, mobility, wound status, and signs of pain or distress. When clinically indicated, supportive treatment was provided according to the approved veterinary care protocol, including gentamicin administration (10 mg/kg) for suspected infection and ketoprofen administration (5 mg/kg) for analgesic and anti-inflammatory management. Animals showing severe wound complications, persistent distress, impaired mobility, or any condition judged to compromise animal welfare despite supportive care were humanely euthanized according to the IACUC-approved humane endpoint criteria.

### 2.2 Localized irradiation

To complete the clinical simulation of the “two-hit” model, localized irradiation (IR) was administered on postoperative day 1 (Figures 2B, 3B,C). Animals were anesthetized using the identical Zoletil and Rompun protocol described above (Section 2.1). Precise anatomical targeting was achieved using an adjustable collimator integrated into an X-RAD 320 biological irradiator (Precision X-Ray, North Branford, CT, United States). The anesthetized rats were positioned radially on the irradiator turntable. The primary radiation field was uniformly configured to encompass the inguinal region and hindlimbs. A single dose of 20 Gy was delivered to this exposed lower body region at a dose rate of approximately 1.7 Gy/min. This standardized irradiation setup was used to provide consistent regional irradiation across animals.

### 2.3 *In Vivo* lymphatic imaging

**FIGURE 4:**
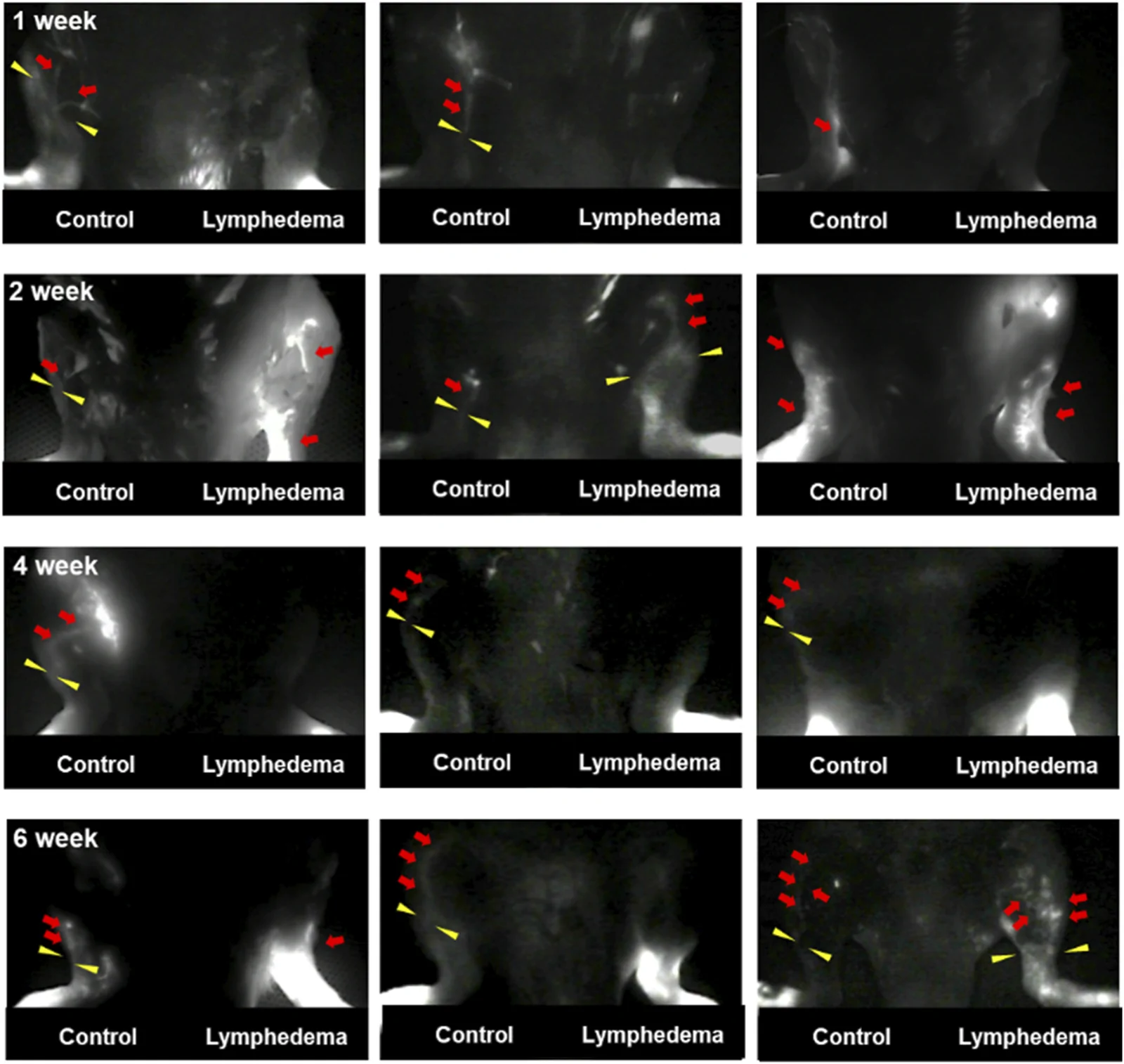
Longitudinal ICG lymphography demonstrating differential lymphatic recovery in paired hindlimbs. Sequential *in vivo* ICG lymphography images obtained at 1, 2, 4, and 6 weeks post-resection. Each row presents an intra-animal comparison between the control hindlimb (CG; left), which underwent circumferential skin resection and localized irradiation, and the lymphedema hindlimb (LG; right), which additionally underwent radical lymphadenectomy. Representative longitudinal images are shown. The complete experimental cohort included six paired animals at weeks 1, 2, and 6 and five paired animals at week 4. Severe dermal backflow and unorganized drainage persisted in the LG, whereas the CG showed gradual restoration of organized linear lymphatic drainage. Yellow arrows indicate severe dermal backflow or lymphatic stasis, and red arrowheads indicate restored linear lymphatic channels. Abbreviations: ICG, indocyanine green; LG, lymphedema group; CG, control group; IR, irradiation; H&E, hematoxylin and eosin staining; IF, Immunofluorescence.

Near-infrared ICG lymphography was utilized to dynamically evaluate real-time lymphatic drainage and functional alterations. Six rats were evaluated at each time point, except at week 4, when one animal was not imaged because of experimental scheduling constraints unrelated to animal condition or outcome; thus, five rats were analyzed at week 4 (Figures 2, 4). Prior to imaging, residual hair surrounding the operative and imaging sites was completely depilated. Under the identical general anesthesia protocol (intramuscular Zoletil and Rompun mixture) described in Section 2.1, a 2.5 mg/mL solution of indocyanine green (ICG; KyungDong Pharmaceutical, Republic of Korea) was administered via subcutaneous injection (0.1 mL) into the dorsal surface of the bilateral hind paws. Spatiotemporal lymphatic flow patterns and dermal backflow severity were captured 10–15 min post-injection using a dedicated near-infrared fluorescence imaging system (Fluobeam, Fluoptics, Grenoble, France). All contrast and brightness adjustments were applied uniformly to the entire image and only for representative visualization. Scoring was performed in a blinded manner using the original image series and predefined criteria. The injection site was excluded from pattern interpretation because of local signal saturation.

To quantitatively evaluate the progression of lymphatic dysfunction, the acquired ICG images were assessed using a modified clinical staging system adapted from (Yamamoto et al., 2011). Dermal backflow severity was classified into four clinically adapted pattern categories (Type I to IIIb) and assigned a corresponding ordinal severity score ranging from 0 to 5. The scoring criteria were defined as follows: scores 0–1 for Type I (linear or near-normal drainage), score 2 for Type II (splash pattern/mild dermal backflow), scores 3–4 for Type IIIa (mixed linear or stardust pattern/moderate dermal backflow), and score 5 for Type IIIb (no organized drainage/severe dysfunction). This classification was used as an ordinal research framework for standardized visual grouping and non-parametric statistical analysis. Formal inter-rater and intra-rater reliability assessments were not performed in the present study.

### 2.4 Histopathological and spatiotemporal morphometric analysis

To evaluate spatiotemporal tissue remodeling, rats were euthanized by CO_2_ inhalation using a gradual-fill method, followed by confirmation of death according to the approved IACUC protocol at designated time points (1, 2, 4, and 6 weeks post-resection) (Figure 2B). Tissue specimens were harvested from functionally distinct anatomical regions distal to the surgical site, specifically the ankle and footpad regions. Samples were fixed in 10% neutral buffered formalin for 24 h at room temperature, followed by decalcification, dehydration, and paraffin embedding. Serial sections (3 μm thick) were prepared and stained with hematoxylin and eosin (H&E) for assessment of dermal thickness and structural remodeling.

Whole-slide images were acquired using a PANNORAMIC 250 Flash III digital slide scanner (3DHISTECH Ltd., Budapest, Hungary) and analyzed using CaseViewer software (3DHISTECH Ltd.). For morphometric analysis, a minimum of three predefined dermal regions of interest were selected from each cross-section. Representative footpad and ankle images were exported from CaseViewer at ×3 and ×5 display magnification, respectively.

Within each predefined region, dermal thickness was measured at five standardized points using CaseViewer software. To minimize the influence of local tissue distortion, sectioning artifacts, and focal irregularities, the highest and lowest measurements were excluded, and the median of the remaining measurements was used for analysis. Each hindlimb contributed a single biological replicate value for statistical analysis.

### 2.5 Multiplex immunofluorescence staining and image quantification

To assess local fibrosis-associated tissue remodeling, multiplex immunofluorescence staining was performed on paraffin-embedded ankle and footpad tissues collected at 1 and 6 weeks post-resection. Three paired animals were analyzed at each time point (n = 3 paired animals per time point), with the control and lymphedema hindlimbs obtained from the same animal.

Tissue sections were cut at a thickness of 3 μm, deparaffinized in xylene for 5 min three times, and rehydrated through graded ethanol solutions (99.9%, 95%, and 70%) to distilled water. Sections were treated with 3% hydrogen peroxide for 10 min and washed three times with phosphate-buffered saline (PBS). Heat-induced antigen retrieval was performed in Tris–EDTA buffer (pH 9.0) at 97 °C for 10 min, followed by blocking with 1% bovine serum albumin in PBS.

Sections were incubated overnight at 4 °C with primary antibodies against collagen III (Novus Biologicals, Cat. No. NBP1-05119, mouse, 1:100), α-smooth muscle actin (α-SMA; LS Bio, Cat. No. LS-B3933, goat, 1:100), and lymphatic vessel endothelial hyaluronan receptor 1 (LYVE-1; Invitrogen, Cat. No. PA116635, rabbit, 1:100). After washing with PBS containing Tween 20, sections were incubated for 1 h at room temperature with the corresponding secondary antibodies: goat anti-mouse Alexa Fluor 488 (Invitrogen, Cat. No. A-11001, 1:200), donkey anti-goat Alexa Fluor 647 (Thermo Fisher Scientific, Cat. No. A-21447, 1:200), and goat anti-rabbit Alexa Fluor 594 (Invitrogen, Cat. No. A-11012, 1:200). Nuclei were counterstained with DAPI.

Fluorescence whole-slide images were acquired at 40× using a KF-FL-120 fluorescence slide scanner (Konfoong Bioinformation Tech Co., Ltd., China). For image presentation, DAPI was displayed in blue, collagen III detected with Alexa Fluor 488 in green, LYVE-1 detected with Alexa Fluor 594 in red-orange, and α-SMA detected with Alexa Fluor 647 in far-red/magenta. All sections were stained using the same protocol and scanned under identical acquisition settings.

Quantitative image analysis was performed using ImageJ software (National Institutes of Health, Bethesda, MD, United States). For each sample, a dermal region of interest was manually delineated within the selected anatomical field and applied consistently to the corresponding fluorescence channels. The epidermis, subcutaneous tissue, and obvious technical artifacts, including tissue folds, tears, and non-anatomical saturated signals, were excluded from the region of interest. Following background correction and thresholding, the positive areas occupied by DAPI, collagen III, and α-SMA were measured.

Collagen III- and α-SMA-positive areas were normalized to the DAPI-positive area using the following calculation: marker-positive area/DAPI-positive area × 100. The normalized value obtained from each hindlimb was treated as one biological replicate. Paired differences were calculated as the normalized value in the LG minus that in the corresponding CG hindlimb. LYVE-1 was included for morphological visualization in the merged images but was not included in the quantitative analysis.

### 2.6 Longitudinal profiling of systemic α-SMA

To characterize early circulating α-SMA responses, serum α-SMA levels were measured on days 1, 2, 5, and 7 post-irradiation (IR) (Figure 3B). To establish accurate, self-controlled baselines while strictly adhering to animal welfare standards (the 3Rs), baseline blood samples were collected from the same animals 1 day before surgery. To minimize cumulative anesthetic toxicity and prevent acute hemodynamic stress during repeated serial sampling, all blood collections were performed under light sedation using a titrated, low-dose mixture of Zoletil and Rompun as described above (Section 2.1). Subsequent longitudinal blood sampling at post-IR time points was conducted. Blood sampling was performed according to predefined welfare criteria. When samples could not be collected, the reason for missing data was recorded and the number of biological replicates at each time point is reported in the corresponding figure legends. The specific number of biological replicates analyzed at each respective time point is detailed in the corresponding figure legends.

Collected blood samples were centrifuged at 2,000 rpm for 40 min at 4 °C to isolate the serum (Singh et al., 2021). Quantification was performed using a Rat α-SMA ELISA Kit (AFG Scientific, Cat. No. EK720946) strictly according to the manufacturer’s protocols. To reduce analytical bias, all assays were executed in a blinded fashion, and optical densities were measured utilizing SpectraMax iD3 and iD5 Multi-Mode Microplate Readers.

### 2.7 Longitudinal profiling of systemic PIIINP

To evaluate circulating PIIINP as an exploratory marker associated with type III collagen formation, serum PIIINP levels were measured during the progressive fibrotic phases on days 7 and 15 post-IR (Figure 2C). Blood collection, mild sedation, and serum isolation procedures were conducted identically to the α-SMA profiling protocol described above. Notably, to maximize data yield while minimizing total animal use in strict accordance with the 3Rs principles, a subset of the banked serum samples acquired during the α-SMA profiling was strategically utilized for PIIINP quantification, supplemented by additional independent samples where necessary.

Serum PIIINP levels were determined using a Rat PIIINP ELISA Kit (Cusabio, Cat. No. CSB-E08096R) according to the manufacturer’s instructions. For samples with PIIINP concentrations falling below the lower limit of detection (LOD), a value equal to half of the lowest standard concentration (LOD/2) was assigned to enable statistical comparison. Optical densities were similarly recorded using the SpectraMax microplate readers in a blinded manner.

### 2.8 Preparation of the conceptual schematic

The conceptual schematic shown in Figure 1 was developed by the authors based on the study design and biological mechanisms reported in the cited literature. ChatGPT (GPT-5.6 Thinking; OpenAI, San Francisco, CA, United States ; accessed July 2026) was used to assist with the initial graphical drafting and organization of the schematic. The resulting illustration was subsequently reviewed and revised by the authors. All labels, directional relationships, and biological interpretations were independently evaluated against the cited literature. Generative AI was not used to generate, alter, or analyze experimental histological, immunofluorescence, lymphographic, or quantitative data.

### 2.9 Statistical analysis

All statistical analyses were performed using GraphPad Prism software (GraphPad Software, Inc., San Diego, CA, United States). Continuous data are presented as the mean ± standard deviation (SD), and ordinal data as the median with interquartile range. Group comparisons were conducted using paired or unpaired Student’s t-tests, with Welch’s t-test applied when unequal variances were detected using the F-test. Paired tests were used for intra-animal comparisons of dermal thickness and DAPI-normalized collagen III- and α-SMA-positive areas, whereas unpaired tests were used for serum marker comparisons between independent CG and LG animals. Intra-animal comparisons of ordinal ICG lymphography scores were performed using the Wilcoxon matched-pairs signed-rank test, while temporal changes in dermal thickness were evaluated using two-way analysis of variance (ANOVA). Longitudinal serum α-SMA data were analyzed using a mixed-effects model with restricted maximum likelihood estimation to accommodate missing values from serial blood collections. Serum PIIINP concentrations were compared between the CG and LG at each time point using unpaired Student’s t-tests or Welch’s t-tests, as appropriate. PIIINP concentrations below the lower limit of detection were assigned a value of LOD/2 for statistical analysis. Statistical significance was defined as p < 0.05 (p < 0.05, *p < 0.01, and **p < 0.001).

## 3 Results

### 3.1 Radical lymphadenectomy prevents recovery of lymphatic drainage and sustains dermal backflow

Longitudinal ICG lymphography was performed at 1, 2, 4, and 6 weeks after surgery (Figure 2B). To systematically evaluate the morphological changes in lymphatic drainage, we established a modified pattern classification system adapted from the clinical staging criteria by Yamamoto et al. (Yamamoto et al., 2011). As illustrated in Figure 2C and summarized in Table 1, dermal backflow patterns were classified into four categories, from Type I to Type IIIb, corresponding to an ordinal score ranging from 0 to 5. This classification enabled both morphological assessment and quantitative comparison of lymphatic dysfunction.

**TABLE 1:** Classification and ordinal scoring of ICG patterns.

Type	Type-level ICG pattern	Ordinal score	Detailed pattern
Type I	Linear/near-normal drainage	0	Normal linear
		1	Aberrant lateral linear
Type II	Mild dermal backflow	2	Splash-like focal dermal backflow
Type IIIa	Moderate dermal backflow	3	Mixed linear + dermal backflow
		4	Stardust-like dermal backflow
Type IIIb	Severe dysfunction	5	No organized drainage

The severity of dermal backflow was classified into four clinical types (Type I–IIIb) for visual grouping, and further detailed into a 0–5 ordinal scale for non-parametric statistical analysis.

Representative images in Figure 4 compare the control hindlimb (CG; circumferential skin resection and irradiation) and the lymphedema hindlimb (LG; radical lymphadenectomy, circumferential skin resection, and irradiation) within the same animal. At 1 and 2 weeks, both hindlimbs exhibited extensive dermal backflow and disrupted lymphatic drainage. At 4 and 6 weeks, however, the drainage patterns gradually diverged. The CG showed partial recovery of organized linear drainage, whereas the LG continued to exhibit stardust-like dermal backflow or an absence of organized lymphatic drainage.

The longitudinal ordinal scores reflected this difference in recovery patterns (Figures 2D,E). Comparisons at individual time points did not reach statistical significance, whereas phase-based analysis showed a clearer difference between the groups. During the acute phase, defined as weeks 1–2, both groups had similarly high severity scores. During the later observation phase, defined as weeks 4–6, severity scores remained significantly higher in the LG than in the CG (p < 0.01; Figures 2D,E).

The distribution of dermal backflow categories showed a similar temporal pattern (Figure 2F). Severe patterns, including Types IIIa and IIIb, were common in both groups during the early postoperative period. By weeks 4 and 6, the CG shifted toward Type I or Type II patterns, whereas all LG hindlimbs remained within the severe categories. At week 6, 50% of the LG hindlimbs were classified as Type IIIa and 50% as Type IIIb. These findings demonstrate persistent lymphatic drainage impairment in the LG throughout the 6-week observation period.

### 3.2 Radical lymphadenectomy produces persistent dermal thickening in the ankle and footpad

**FIGURE 5:**
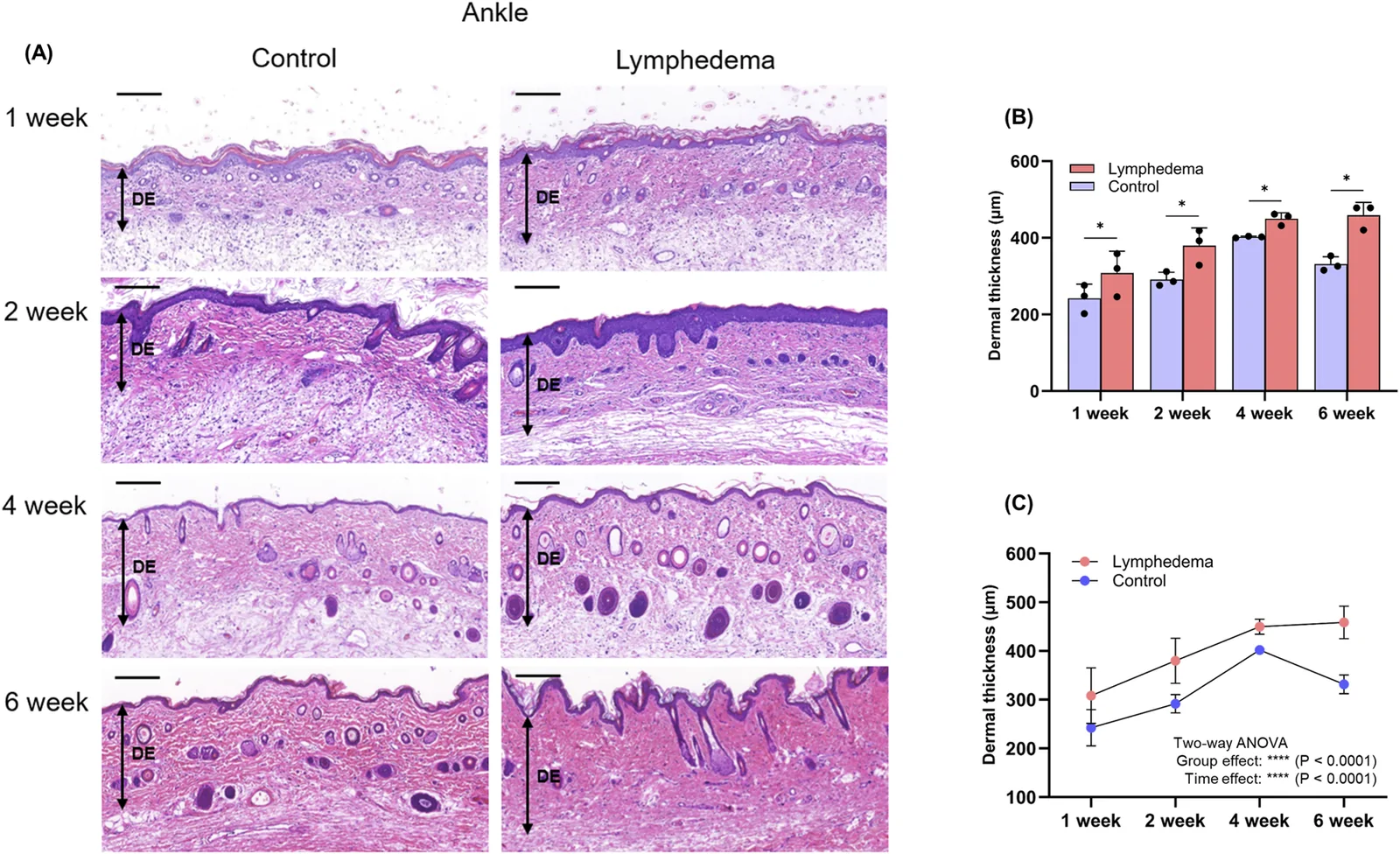
Paired histological assessment of dermal thickening in the ankle region. **(A)** Representative H&E-stained sections of the ankle region from the CG hindlimb and LG hindlimb at 1, 2, 4, and 6 weeks post-resection. Representative images were exported from CaseViewer at ×5 display magnification. Scale bar = 200 μm. **(B)** Quantitative comparison of dermal thickness between the paired CG and LG hindlimbs at each time point. Comparisons were performed using a paired Student’s t-test **(C)** Temporal profiles of dermal thickness in the CG and LG. The effects of group and time were evaluated using two-way ANOVA. Data are presented as the mean ± SD. Each time point included three paired animals (n = 3 per time point). The same animals contributed the ankle and footpad samples shown in Figures 5, 6. *p < 0.05 and ****p < 0.0001. Abbreviations: CG, control group; DE, dermis; H&E, hematoxylin and eosin; LG, lymphedema group.

**FIGURE 6:**
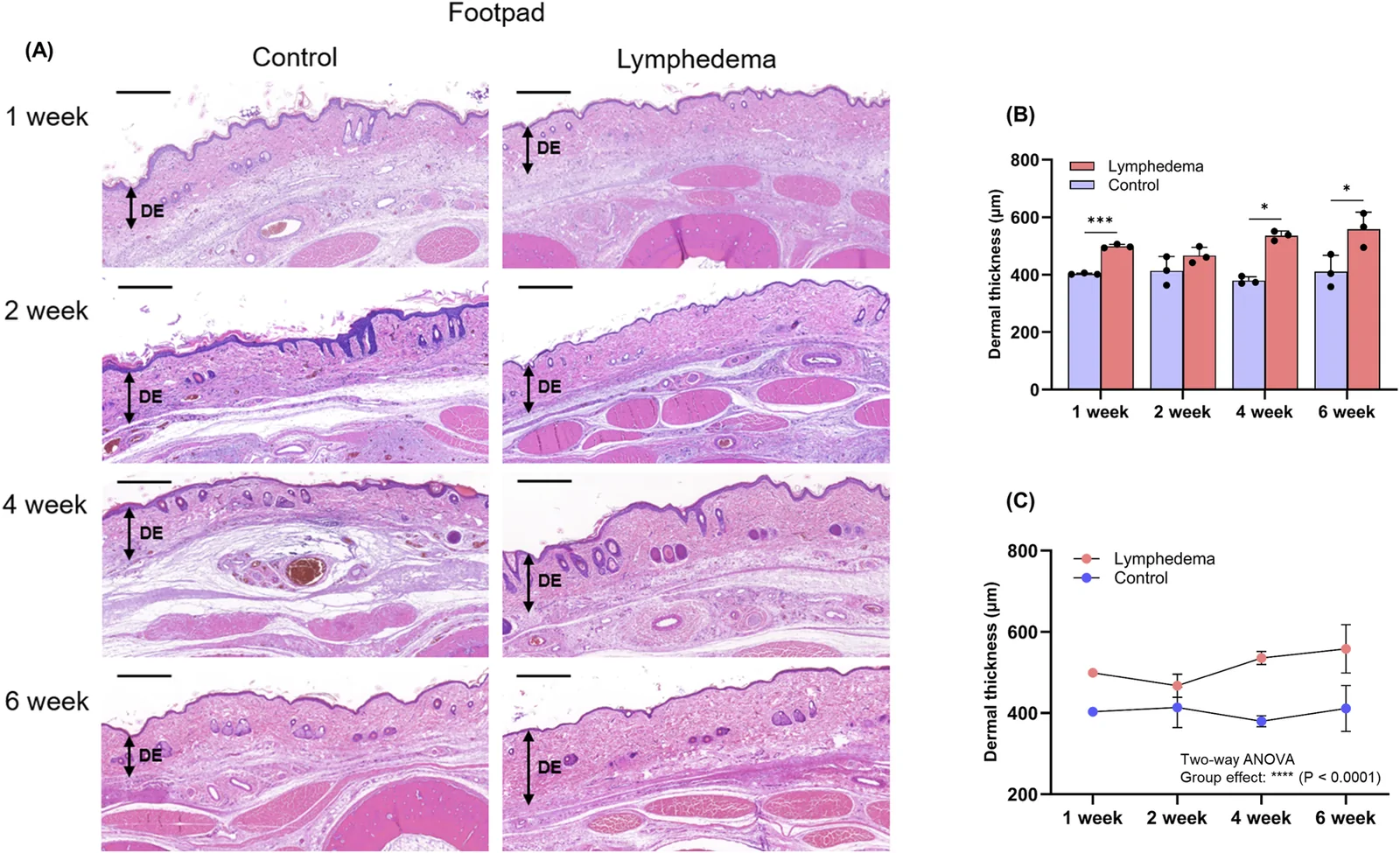
Paired histological assessment of dermal thickening in the footpad region. **(A)** Representative H&E-stained sections of the footpad region from the CG and LG at 1, 2, 4, and 6 weeks post-resection. Representative images were exported from CaseViewer at ×3 display magnification. Scale bar = 400 μm. **(B)** Quantitative comparison of dermal thickness between the paired CG and LG hindlimbs at each time point. Comparisons were performed using a paired Student's tt-test. **(C)** Temporal profiles of dermal thickness in the CG and LG. The effects of group and time were evaluated using two-way ANOVA. Data are presented as the mean ± SD. Each time point included three paired animals (n = 3 per time point). The same animals contributed the ankle and footpad samples shown in Figures 5, 6. *p < 0.05, and ***p < 0.001, and ****p < 0.0001. Abbreviations: CG, control group; DE, dermis; H&E, hematoxylin and eosin; LG, lymphedema group.

Histological analysis was performed to evaluate dermal structural changes in the ankle and footpad regions (Figures 5, 6). At the ankle, dermal thickness was greater in the LG than in the corresponding CG at 1, 2, 4, and 6 weeks after surgery (Figure 5B). The CG showed a transient increase in dermal thickness, with the greatest thickness observed at 4 weeks, followed by a reduction at 6 weeks. In contrast, increased dermal thickness persisted in the LG throughout the observation period. At 6 weeks, the ankle dermis remained thicker in the LG than in the CG (Figure 5).

A similar between-limb difference was observed in the footpad. The LG showed greater dermal thickness than the CG at all evaluated time points (Figure 6). Although the magnitude of dermal thickening varied across time points, the difference between the LG and CG remained evident at 6 weeks. These findings indicate that radical lymphadenectomy was associated with sustained dermal thickening in both the ankle and footpad regions.

### 3.3 Region- and time-dependent changes in collagen III and α-SMA signals

**FIGURE 7:**
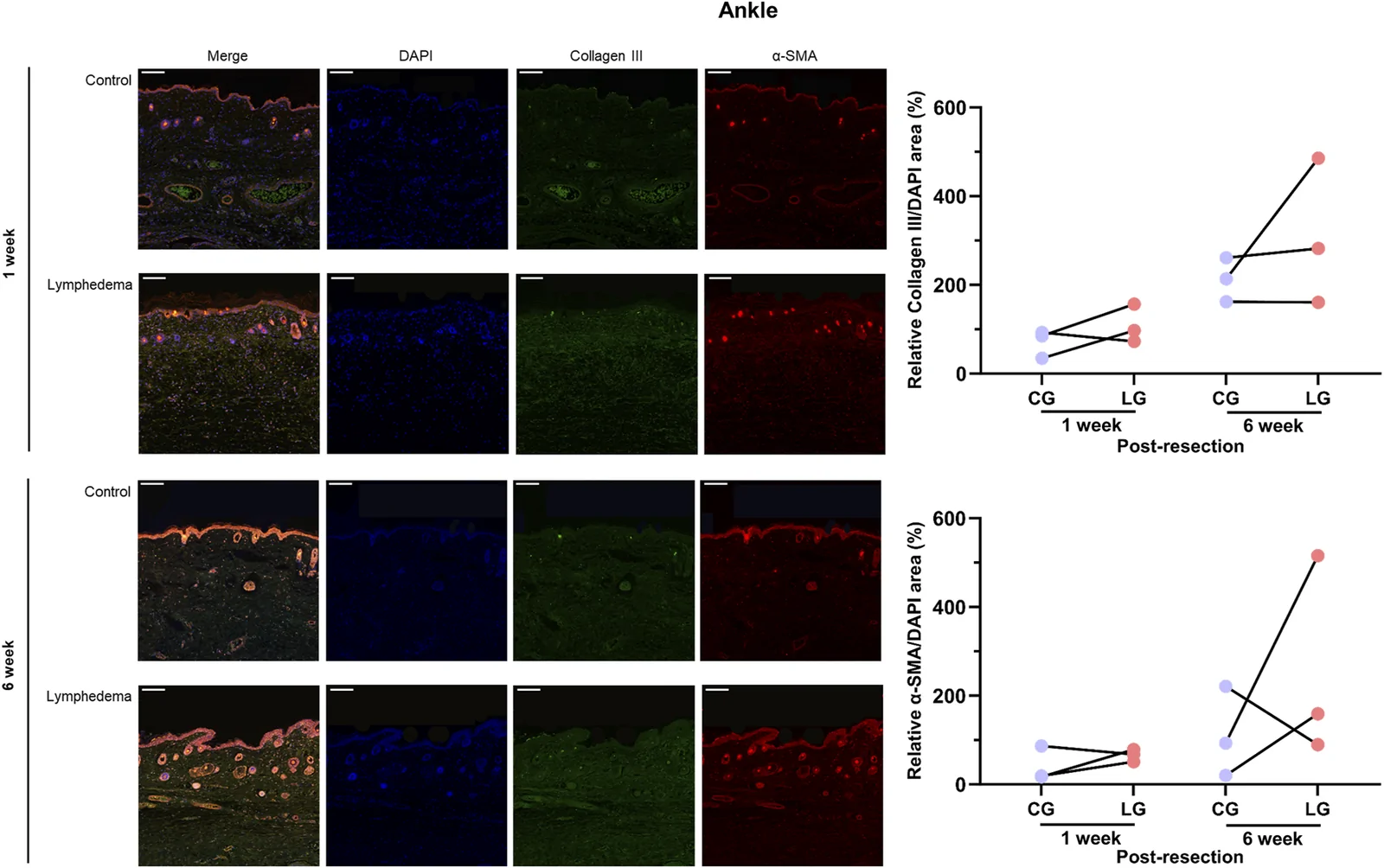
Paired immunofluorescence assessment of collagen III and α-SMA in the ankle dermis. **(A)** Representative immunofluorescence images of CG and LG hindlimbs at 1 and 6 weeks post-resection. Merged images include DAPI (blue), collagen III (green), LYVE-1 (red-orange), and α-SMA (far-red/magenta), with the corresponding DAPI, collagen III, and α-SMA single-channel images shown separately. LYVE-1 was displayed only in the merged images and was not included in the quantitative analysis. Whole-slide images were acquired at 40×, and representative fields are shown at ×5.5 display magnification; scale bar, 150 μm. **(B)** Paired quantification of DAPI-normalized collagen III-positive area. **(C)** Paired quantification of DAPI-normalized α-SMA-positive area. Normalized values were calculated as the marker-positive area divided by the DAPI-positive area × 100. Lines connect the CG and LG hindlimbs from the same animal. Each time point included three paired animals (n = 3 per time point). Paired comparisons were performed using a paired Student's tt-test; none of the comparisons reached statistical significance. The same animals contributed the footpad samples shown in Figure 8. Abbreviations: α-SMA, alpha-smooth muscle actin; CG, control group; DAPI, 4',6-diamidino-2-phenylindole; LG, lymphedema group; LYVE-1, lymphatic vessel endothelial hyaluronan receptor 1.

**FIGURE 8:**
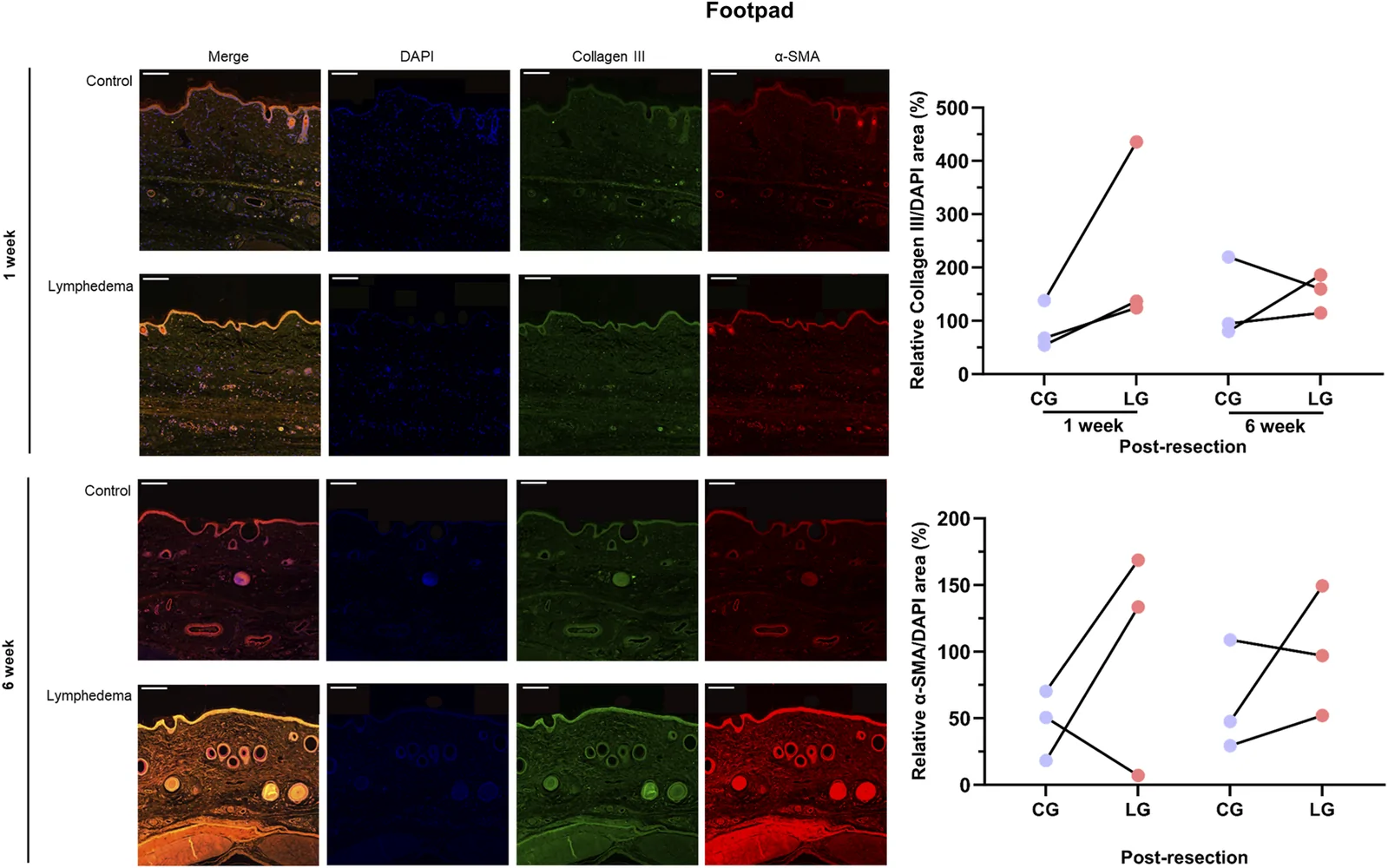
Paired immunofluorescence assessment of collagen III and α-SMA in the footpad dermis. (A) Representative immunofluorescence images of CG and LG hindlimbs at 1 and 6 weeks post-resection. Merged images include DAPI (blue), collagen III (green), LYVE-1 (red-orange), and α-SMA (far-red/magenta), with the corresponding DAPI, collagen III, and α-SMA single-channel images shown separately. LYVE-1 was displayed only in the merged images and was not included in the quantitative analysis. Whole-slide images were acquired at 40×, and representative fields are shown at ×6.0 display magnification; scale bar, 150 μm. (B) Paired quantification of DAPI-normalized collagen III-positive area. (C) Paired quantification of DAPI-normalized α-SMA-positive area. Normalized values were calculated as the marker-positive area divided by the DAPI-positive area × 100. Lines connect the CG and LG hindlimbs from the same animal. Each time point included three paired animals (n = 3 per time point). Paired comparisons were performed using a paired Student's t-test; none of the comparisons reached statistical significance. The same animals contributed the ankle samples shown in Figure 7. Abbreviations: α-SMA, alpha-smooth muscle actin; CG, control group; DAPI, 4',6-diamidino-2-phenylindole; LG, lymphedema group; LYVE-1, lymphatic vessel endothelial hyaluronan receptor 1.

To assess local fibrosis-associated tissue changes, collagen III- and α-SMA-positive areas were quantified in the ankle and footpad at 1 and 6 weeks after surgery (Figures 2, 7, 8). Measurements were obtained from paired control and lymphedema hindlimbs within the same animal. The paired difference was calculated as the normalized value in the LG minus the corresponding value in the CG.

At the ankle, collagen III-positive area was higher in the LG in two of three paired animals at both 1 and 6 weeks (Figure 7 and Table 2). The mean paired difference was 37.97 ± 50.47 percentage points at 1 week and 97.24 ± 151.84 percentage points at 6 weeks. The large variation at 6 weeks was primarily associated with a marked increase in one animal. The α-SMA-positive area was higher in the LG in two of three paired animals at both 1 and 6 weeks. The corresponding mean paired differences were 24.81 ± 41.00 percentage points at 1 week and 143.25 ± 276.80 percentage points at 6 weeks, respectively (Table 3). Considerable variability was observed at 6 weeks, with one animal showing a decrease and another showing a marked increase.

**TABLE 2:** Paired differences in DAPI-normalized collagen III-positive area.

Region and time	Rat 1 Δ	Rat 2 Δ	Rat 3 Δ	Mean ± SD	Direction
Ankle, 1 week	−20.08	71.49	62.50	37.97 ± 50.47	2/3 increased
Ankle, 6 weeks	272.12	−1.16	20.78	97.24 ± 151.84	2/3 increased
Footpad, 1 week	82.81	297.53	56.56	145.63 ± 132.20	3/3 increased
Footpad, 6 weeks	−59.88	105.64	19.97	21.91 ± 82.77	2/3 increased

Δ was calculated as the DAPI-normalized collagen III-positive area in the LG, minus that in the paired CG, hindlimb and is expressed in percentage points. Positive values indicate LG > CG., Each condition included three paired animals (n = 3). None of the paired comparisons reached statistical significance. CG, control group; DAPI, 4′,6-diamidino-2-phenylindole; LG, lymphedema group.

**TABLE 3:** Paired differences in DAPI-normalized α-SMA-positive area.

Region and time	Rat 1 Δ	Rat 2 Δ	Rat 3 Δ	Mean ± SD	Direction
Ankle, 1 week	−19.33	61.70	32.05	24.81 ± 41.00	2/3 increased
Ankle, 6 weeks	−131.20	138.61	422.33	143.25 ± 276.80	2/3 increased
Footpad, 1 week	115.22	98.53	−43.55	56.73 ± 87.25	2/3 increased
Footpad, 6 weeks	−11.85	101.83	22.61	37.53 ± 58.29	2/3 increased

Δ was calculated as the DAPI-normalized α-SMA-positive area in the LG, minus that in the paired CG, hindlimb and is expressed in percentage points. Positive values indicate LG > CG., Each condition included three paired animals (n = 3). None of the paired comparisons reached statistical significance. α-SMA, alpha-smooth muscle actin; CG, control group; DAPI, 4′,6-diamidino-2-phenylindole; LG, lymphedema group.

In the footpad, collagen III-positive area was higher in the LG in all three paired animals at 1 week and in two of three animals at 6 weeks (Figure 8 and Table 2). The mean paired difference was 145.63 ± 132.20 percentage points at 1 week and 21.91 ± 82.77 percentage points at 6 weeks. The α-SMA-positive area was higher in the LG in two of three paired animals at both 1 and 6 weeks. The corresponding mean paired differences were 56.73 ± 87.25 percentage points at 1 week and 37.53 ± 58.29 percentage points at 6 weeks, respectively (Table 3).

None of the paired comparisons reached statistical significance, and considerable inter-animal variability was observed, particularly for collagen III and ankle α-SMA at 6 weeks. Nevertheless, the paired data showed directional increases in collagen III and α-SMA signals in several region- and time-specific comparisons. These findings provide local tissue-level evidence of fibrosis-associated changes in subsets of the LG samples but do not demonstrate uniform increases across all anatomical regions, time points, or animals.

### 3.4 Circulating α-SMA was elevated at selected early time points, whereas PIIINP differences were not significant

To evaluate circulating fibrosis-associated markers after the intervention, serum α-SMA and PIIINP levels were measured in separate cohorts of animals. Unlike the paired tissue analyses, the CG and LG serum samples were obtained from different animals (Figure 3A).

Serum α-SMA was measured before surgery and at 1, 2, 5, and 7 days after irradiation (Figure 3B). Both groups showed an increase in circulating α-SMA at day 1 relative to the preoperative baseline (Figures 3D–F). The LG exhibited higher α-SMA levels than the CG at day 1 and day 7. At days 2 and 5, the differences between the groups did not reach statistical significance. These findings demonstrate differential circulating α-SMA levels at selected time points but do not establish that the changes were specific to lymphedema rather than to surgery, irradiation, or tissue repair.

Serum PIIINP was measured at days 7 and 15 to evaluate systemic changes associated with type III collagen turnover (Figure 3C). Values below the assay limit of detection were assigned a value of LOD/2 for statistical analysis. PIIINP levels were numerically higher in the LG than in the CG at both time points; however, the between-group differences were not statistically significant at day 7 (p = 0.2323) or day 15 (p = 0.2113). Thus, the PIIINP results represent a non-significant exploratory trend rather than evidence of a confirmed systemic increase.

The local IF and serum analyses were performed in different animal cohorts, and subject-level correlations between tissue collagen III or α-SMA signals and circulating marker concentrations were not assessed.

## 4 Discussion

The development of clinically relevant secondary lymphedema models remains challenging because lymphatic injury in rodents is frequently followed by collateral lymphatic regeneration and partial spontaneous recovery (Frueh et al., 2016; Hsu et al., 2022). Despite advances in microsurgical and pharmacological treatments, clinical translation remains limited by the lack of reproducible preclinical models and standardized outcome measures (O'Donnell et al., 2020; Will et al., 2022). In the present study, we established a rat hindlimb model by combining radical lymphadenectomy, circumferential skin resection, and localized irradiation and evaluated its progression using complementary functional, histological, tissue-level, and circulating endpoints. The principal strength of this study is the integration of longitudinal ICG lymphography, paired histological evaluation of two anatomically distinct regions, local immunofluorescence analysis of collagen III and α-SMA, and serial assessment of circulating fibrosis-associated markers. Together, these analyses demonstrate that radical lymphatic ablation prevented functional recovery and was accompanied by sustained dermal thickening and region-dependent changes in fibrosis-associated tissue signals.

Previous rat hindlimb models have used lymph node excision, circumferential soft-tissue disruption, and irradiation in various combinations to overcome rapid lymphatic regeneration and establish sustained lymphatic dysfunction (Frueh et al., 2016; Hsu et al., 2022; Buntinx et al., 2022; To et al., 2010; Mitra et al., 2016; Huang et al., 2021; Had et al., 2017). Removal of the inguinal and popliteal lymph nodes combined with irradiation has been reported to produce persistent lower-limb lymphedema in rodents’ model (Buntinx et al., 2022; To et al., 2010; Mitra et al., 2016), whereas other models have incorporated circumferential skin and subcutaneous tissue excision to interrupt superficial collateral drainage (Huang et al., 2021; Had et al., 2017). Experimental comparisons of surgery and irradiation have also shown that their combination produces more sustained lymphatic and tissue abnormalities than either intervention alone (Buntinx et al., 2022; To et al., 2010; Mitra et al., 2016). These findings provide a biological and technical rationale for the combined approach used in the present study.

An important feature of our experimental design was that both the CG and LG underwent circumferential skin resection and localized irradiation, while radical lymphadenectomy was performed only in the LG. Consequently, early abnormalities shared by the two groups were likely influenced by postoperative injury and irradiation, whereas the subsequent divergence between the paired hindlimbs reflected the additional effect of extensive lymphatic ablation. At 1 and 2 weeks, both groups exhibited severe dermal backflow, consistent with acute disruption of lymphatic drainage after surgery and irradiation. At 4 and 6 weeks, however, the CG demonstrated partial restoration of organized linear drainage, whereas the LG continued to exhibit severe dermal backflow and an absence of organized lymphatic channels. The significantly higher phase-level severity score in the LG during weeks 4–6 and the persistence of Type IIIa or IIIb patterns in all LG hindlimbs at week 6 indicate that radical lymphadenectomy limited spontaneous lymphatic recovery beyond the effects of skin resection and irradiation alone.

ICG lymphography is particularly relevant to the translational assessment of this model because clinical lower-extremity lymphedema is characterized by a transition from organized linear drainage to splash, stardust, and diffuse dermal backflow patterns as lymphatic dysfunction progresses (Yamamoto et al., 2011). Our modified ordinal system retained this established morphological framework while subdividing severe unorganized drainage patterns to provide greater resolution within the rat hindlimb. Although the adapted categories require further validation before direct comparison with clinical stages, the persistent stardust-like and unorganized patterns observed in the LG reproduce important functional imaging features of advanced lymphatic insufficiency. The longitudinal design is also important because the distinction between temporary postoperative lymphatic disruption and persistent dysfunction became evident only during the later observation period.

The functional divergence identified by ICG lymphography was accompanied by sustained dermal structural changes. Dermal thickness was greater in the LG than in the paired CG at the ankle and footpad throughout the observation period. The inclusion of both regions allowed the model to capture spatially distributed tissue remodeling rather than changes confined to a single sampling site. The ankle represented the more proximal sampling region, whereas the footpad reflected distal tissue exposed to impaired lymphatic clearance. Persistent thickening at both sites therefore supports the presence of spatially distributed consequences of lymphatic obstruction, consistent with the structural changes observed in clinical lymphedema (Buntinx et al., 2022; To et al., 2010). Nevertheless, dermal thickness measured using H&E staining does not independently demonstrate collagen deposition or myofibroblast activation. For this reason, the addition of tissue-level collagen III and α-SMA analysis substantially strengthens the pathological characterization of the model.

Collagen III and α-SMA were selected as complementary indicators of fibrosis-associated tissue remodeling. Increased collagen III expression has been reported in fibrotic skin obtained from patients with lower-extremity lymphedema (Karayi et al., 2020). Experimental studies have also linked lymphedematous fibrosis to increased extracellular matrix deposition and TGF-β1 signaling. Inhibition of TGF-β1 in experimental lymphedema reduced extracellular matrix accumulation and tissue fibrosis while improving collateral lymphatic formation, supporting the close relationship between fibrotic remodeling and persistent lymphatic dysfunction (Baik et al., 2022). These observations provide biological context for evaluating collagen III and α-SMA in the affected dermis.

In the present study, paired IF analysis demonstrated region- and time-dependent differences in fibrosis-associated signals. At the ankle, collagen III-positive area was higher in two of three LG hindlimbs at both 1 and 6 weeks. The α-SMA-positive area was also higher in two of three LG hindlimbs at both time points. In the footpad, collagen III-positive area was higher in all three LG hindlimbs at 1 week and in two of three LG hindlimbs at 6 weeks, whereas α-SMA-positive area was higher in two of three LG hindlimbs at both 1 and 6 weeks. None of the paired IF comparisons reached statistical significance, and substantial inter-animal variation was observed, particularly for collagen III and ankle α-SMA at 6 weeks. Accordingly, these findings should not be interpreted as uniform upregulation across every anatomical site, time point, or animal. Rather, the directional paired changes provide tissue-level evidence that radical lymphatic ablation was associated with fibrosis-related remodeling in specific spatial and temporal contexts.

The heterogeneity of the IF findings is biologically plausible because tissue remodeling does not necessarily progress uniformly throughout a lymphedematous limb. Differences in local lymphatic anatomy, mechanical loading, proximity to the surgical field, collateral drainage, skin architecture, and the temporal balance between active repair and established extracellular matrix deposition may produce distinct responses in the ankle and footpad. The α-SMA findings did not show a uniform anatomical or temporal pattern, with two of three paired animals showing higher values in the LG at each evaluated region and time point. In particular, the large mean paired difference in ankle α-SMA at 6 weeks was influenced by marked inter-animal variability, including a decrease in one animal and a pronounced increase in another, and therefore should not be interpreted as consistent late proximal activation. In the footpad, collagen III was higher in all three LG hindlimbs at 1 week, whereas α-SMA was higher in two of three paired animals, suggesting a predominantly but not uniformly positive early distal response. These interpretations remain hypotheses because cell-specific co-localization and longitudinal tissue sampling were not performed. Nevertheless, the paired analysis reveals information that would have been obscured by group means alone and supports the use of anatomically defined tissue sampling in future therapeutic studies.

α-SMA should also be interpreted as a fibrosis-associated marker rather than a marker specific to lymphedema. α-SMA is expressed by activated myofibroblasts during tissue repair but can also be detected in vascular smooth muscle cells and other contractile structures. Experimental wound-healing studies have demonstrated transient α-SMA expression during granulation tissue formation, confirming that α-SMA can reflect general tissue injury and repair as well as pathological fibrosis (Darby et al., 1990). Therefore, the increase in dermal α-SMA-positive area observed in subsets of the LG samples does not independently identify a lymphedema-specific cell population. Its relevance in this study arises from its interpretation together with persistent lymphatic dysfunction, dermal thickening, and collagen III-associated remodeling in the affected hindlimb.

The tissue findings also provide a biological framework for interpreting the circulating marker results. Serum α-SMA was higher in the LG than in the CG at days 1 and 7 after irradiation, whereas the remaining time-point comparisons did not reach statistical significance. Because the CG underwent the same circumferential skin resection and irradiation, these shared interventions cannot fully explain the between-group differences. The greater α-SMA response in the LG at selected time points suggests that radical lymphadenectomy may have altered the magnitude or duration of the systemic tissue-repair response. However, the serum and tissue analyses were performed in separate animal cohorts, and direct subject-level correlations between dermal α-SMA expression and circulating α-SMA concentrations could not be assessed. The results therefore support circulating α-SMA as a potential fibrosis-associated systemic readout in this model rather than as a validated or lymphedema-specific biomarker. The combined functional imaging, regional histological, local IF, and circulating marker findings provide complementary biological context but do not establish a direct relationship between local tissue remodeling and serum α-SMA. Future studies should examine these relationships within the same experimental cohort.

This distinction is important because the potential significance of circulating α-SMA is not based solely on its systemic elevation. Rather, it should be interpreted in the context of persistent lymphatic dysfunction on ICG lymphography, sustained dermal thickening, and directional α-SMA and collagen III changes observed in subsets of the affected hindlimb tissues. This multimodal context increases the biological plausibility that the serum response was associated with the more extensive tissue injury and remodeling produced by radical lymphatic ablation. Nevertheless, serum α-SMA may still reflect myofibroblast activation associated with surgery, irradiation, wound repair, or other injured tissues. Future studies will therefore be required to determine whether its concentration correlates quantitatively with local fibrosis burden, ICG severity, treatment response, or longer-term disease progression.

Serum PIIINP should be interpreted more cautiously. PIIINP is released during type III collagen turnover and has been evaluated as a systemic fibrosis-associated marker in fibroproliferative and wound-healing conditions. Serum PIIINP can be altered after surgical trauma and burn injury, indicating that it is not specific to lymphatic disease (Ulrich et al., 2003; Tomas et al., 1993). In the present study, PIIINP concentrations were numerically higher in the LG than in the CG at days 7 and 15; however, neither difference reached statistical significance (p = 0.2323 and p = 0.2113, respectively), and several measurements were below the assay limit of detection. Thus, PIIINP cannot be considered a confirmed systemic indicator of lymphedema progression based on the current data. Its directional change, together with collagen III-associated tissue changes, provides a hypothesis-generating observation that warrants assessment in a larger cohort using an assay optimized for the expected concentration range.

Taken together, the serum findings indicate that α-SMA and PIIINP have different levels of evidentiary support. Circulating α-SMA demonstrated significant group differences at selected early time points and therefore represents the stronger candidate for further evaluation. PIIINP showed only a non-significant trend and should remain an exploratory collagen-turnover marker. Neither marker should currently be described as specific for lymphedema. Instead, they may provide complementary systemic information when interpreted together with regional functional imaging and tissue-level analyses. Establishing such a composite endpoint may be more informative than attempting to use an isolated circulating marker to diagnose or stage a predominantly regional disease.

Relative to approaches that validate rodent lymphedema primarily through limb enlargement, bioimpedance, or endpoint lymphatic imaging, the present study provides a broader assessment of disease development. Published rat models have demonstrated that lymphadenectomy combined with irradiation can produce persistent lower-limb abnormalities (Frueh et al., 2016; Hsu et al., 2022; Buntinx et al., 2022; To et al., 2010; Mitra et al., 2016; Huang et al., 2021; Had et al., 2017); however, the present analysis adds serial characterization of dermal backflow, paired assessment of proximal and distal dermal thickness, spatial IF analysis of collagen III and α-SMA, and circulating marker profiling. The value of this design lies not in any single measurement but in the concordance and complementarity of the measurements. Functional impairment was documented longitudinally, structural remodeling was confirmed at multiple anatomical sites, fibrosis-associated signals were assessed directly within the tissue, and systemic marker responses were evaluated as potential minimally invasive adjuncts.

The bilateral paired design further strengthens the regional analyses. Establishing the CG and LG within the same animal reduces variability related to age, body weight, baseline lymphatic anatomy, and individual healing capacity. Furthermore, because both limbs received circumferential skin resection and irradiation, the design provides a stringent comparison for identifying the incremental effects of radical lymphadenectomy. This does not completely eliminate the influence of systemic wound healing or radiation exposure, particularly for serum measurements performed in separate animals, but it improves attribution of the persistent regional differences to the severity of lymphatic disruption.

Several limitations should be considered. First, the 6-week observation period demonstrates persistence beyond the acute postoperative phase but does not fully characterize the long-term or end-stage course of the model. Longer follow-up will be required to determine whether the functional and tissue changes remain stable, continue to progress, or undergo delayed recovery. Second, the IF analysis included three paired animals per condition and showed region-dependent variability. The paired design and presentation of individual trajectories improve transparency and reduce the influence of inter-animal variability, but larger cohorts are required to confirm the observed directional patterns. Finally, α-SMA is not specific to myofibroblasts or lymphatic fibrosis, and PIIINP can be influenced by systemic collagen turnover and wound healing. Future studies should further define the cellular sources of α-SMA through fibroblast-specific co-localization and validate extracellular matrix deposition using complementary fibrosis-specific analyses, such as Picrosirius red staining or collagen protein quantification.

Despite these limitations, the present model offers several practical and translational advantages. The size of the rat hindlimb facilitates repeated lymphatic imaging, anatomically defined tissue sampling, and microsurgical manipulation. The model can therefore support preclinical testing of lymphovenous anastomosis, vascularized lymph node transfer, biomaterial-based lymphatic reconstruction, antifibrotic agents, and regenerative therapies. Therapeutic efficacy can be assessed using a multimodal framework that includes restoration of organized lymphatic drainage, reduction of dermal thickening, modulation of local fibrosis-associated signals, and changes in exploratory circulating markers.

## 5 Conclusion

In conclusion, we established a rat model of secondary lymphedema by combining radical lymphadenectomy, circumferential skin resection, and localized irradiation. Longitudinal ICG lymphography demonstrated persistent dermal backflow and impaired recovery of organized lymphatic drainage, while paired histological analyses showed sustained dermal thickening in both the ankle and footpad. Collagen III and α-SMA IF analyses revealed directional, region- and time-dependent fibrosis-associated tissue changes, although these patterns were variable among animals and did not reach statistical significance. Circulating α-SMA showed significant differences at selected time points and may provide a complementary systemic readout, whereas PIIINP remained an exploratory collagen-turnover marker. Together, the integration of functional imaging, regional histology, tissue-level IF, and circulating marker analysis provides a multimodal preclinical framework for investigating secondary lymphedema progression and evaluating microsurgical, pharmacological, and regenerative therapies.

## Data Availability

The original contributions presented in the study are included in the article/Supplementary Material, further inquiries can be directed to the corresponding authors.
